# Cerebellar ataxia and intrathecal baclofen therapy: Focus on patients´ experiences

**DOI:** 10.1371/journal.pone.0180054

**Published:** 2017-06-27

**Authors:** Shala Ghaderi Berntsson, Anne-Marie Landtblom, Gullvi Flensner

**Affiliations:** 1 Department of Neuroscience, Neurology, Uppsala University, University Hospital, Uppsala, Sweden; 2 Department of Clinical and Experimental Medicine, Neurology, Medical Faculty, University of Linköping, Linköping, Sweden; 3 Department of Health Sciences, University West, Trollhättan, Sweden; University of Pennsylvania Perelman School of Medicine, UNITED STATES

## Abstract

Elucidating patients´ experiences of living with chronic progressive hereditary ataxia and the symptomatic treatment with intrathecal baclofen (ITB) is the objective of the current study. A multicenter qualitative study with four patients included due to the rare combination of hereditary ataxia and ITB therapy was designed to elucidate participants’ experiences through semi-structured interviews. The transcribed text was analyzed according to content analysis guidelines. Overall we identified living in the present/ taking one day at a time as the main theme covering the following categories: 1) Uncertainty about the future as a consequence of living with a hereditary disease; The disease; 2) Impact on life as a whole, 3) Influence on personal life in terms of feeling forced to terminate employment, 4) Limiting daily activities, and 5) ITB therapy, advantages, and disadvantages. Uncertainty about the future was the category that affected participants’ personal life, employment, and daily activities. The participants’ experience of receiving ITB therapy was expressed in terms of improved quality of life due to better body position and movement as well as better sleep and pain relief.

## Introduction

Living with a chronic progressive neurologic disease such as hereditary ataxia has a great impact on the quality of life of patients. The molecular, pathologic and clinical features of hereditary ataxias have been extensively described in the literature [1–5]. However, qualitative studies in hereditary ataxias are sparse [6, 7] and the patients’ experiences of intrathecal baclofen (ITB) therapy, a rare treatment in this patient category have not been reported earlier.

Friedreich Ataxia (FRDA) is the most common form of autosomal recessive cerebellar ataxia [8], whereas spinocerebellar ataxia type 3 (SCA3)/Machado Joseph disease and spinocerebellar ataxia type 7 (SCA7) are inherited in an autosomal dominant manner [2, 9]. The predominant clinical features include cerebellar ataxia as the initial symptom and variable pyramidal degeneration leading to weakness and lower limb spasticity [5].

Spastic-ataxias constitute a small group of genetic disorders that present with the clinical features of ataxia and pyramidal signs.

The pyramidal degeneration causes spasticity and painful spasms in lower limbs in patients with hereditary ataxia affecting patient´s daily activity as well as sleep quality. Systemic pharmacologic therapy is preferred to local treatment in conditions in which muscle over activity involves several areas such as inherited ataxia. The mode of action of most available antispastic drugs (baclofen, diazepam, dantrolene sodium, and tizanidine) is by alteration of transmitters or neuromodulators in the CNS. Baclofen is a structural GABA_B_ agonist, being the drug of choice for many decades. However, orally given baclofen is associated with a wide range of adverse effects related to central depression such as sedation, fatigue, impaired attention and memory, nausea, and dizziness as well as respiratory depression [10]. ITB therapy is delivered via a closed system using an implanted pump into the abdomen and a catheter passing through the spinal CSF space [11]. The treatment is highly effective in reducing spasticity without the systemic side effects of oral medications, which means a higher tolerability in patients who are already suffering from a high burden of disability and in need of assistance for their daily life.

The positive effects of the ITB therapy in relieving painful spasms in patients with inherited ataxia was reported in two case studies, but the patients’ own experiences were not included [12, 13].

The existing amount of qualitative research in the field of inherited ataxia is limited. Exploring the process of diagnosing progressive ataxia from the patients’ perspective and their experiences revealed uncertainty around labeling, treatment, and prognosis at the first step and patient expectations in relation to a treatment or cure in the next. Receiving practical help such as physical and other therapies was highly appreciated [6, 7]. A few studies evaluated different physiotherapeutic approaches in FRDA and tele-coaching intervention in spinocerebellar disorders [14–16].

Qualitative studies in other patient categories with chronic conditions such as epilepsy described patients´ experiences in terms of difficulty with personal development, limitation of personal potential and responsibilities, feeling of alienation, relationships with family and friends, dependency on other people, and meeting ignorance in society [17]. Patients with Multiple sclerosis described how they learned to live with an uncertain future from the moment they received the diagnosis and how they tried to cope with the impact the MS symptoms on their daily lives by taking one day at a time [18].

The aim of this study was to elucidate and better understand patients´ experiences of living with hereditary ataxia in general and to include their experiences of ITB therapy more specifically. The qualitative design of the study with open-ended questions at first allows the patients to share their unique experiences each. In this context, we may extend our knowledge of how patients with the incurable hereditary ataxia managed to face the diagnosis, and to cope with the impact of the disease on the personal, social and family life, and finally to explore the significance of symptomatic ITB therapy in their daily lives.

## Material and methods

### Design

Taking into account the rarity of the combination of hereditary ataxia and ITB therapy, we were able to include five patients through the whole Scandinavia. Despite announcing the study in a Scandinavian ITB therapy meeting in September 2014 in Stockholm and email contact with responsible physicians in Norway, Denmark, Finland, and other centers in Sweden, we could not identify more participants. The study was therefore ended up including two centers out of five in Sweden (Uppsala and Linköping).

A qualitative design approach was selected to cover the experiences of the participants of living with cerebellar ataxia and the new treatment for dealing with spasticity and painful spasms.

A total of five patients were included in the study, three patients from the Neurology Department in Uppsala and two from the Rehabilitation Department in Linköping. The study was approved as a multicenter study by the institutional review board in Uppsala (Dnr 2014/ 315). An information letter about the study was sent to the patients by a research nurse, in which they were informed that the participation in the study is voluntary and it will not alter the medical management and care for the patient. They have also been given the opportunity to pose the questions, and were informed that they could withdraw from the study at any time without any explanation. This information letter is included in the ethical application for the study.

A verbal and written informed consent was obtained from the patients prior to participation. However, one patient that was initially included in the study, passed away shortly before the interview. Thus, in total, the study is based on interviews of four patients. The inclusion criteria and the important characteristics of the sample were patients with a diagnosis of inherited cerebellar ataxia and ITB therapy and the selection was purposive. There was no dropout or refusal to participate in the study. Three participants 2, 3, and 4 knew one of the authors (SGB), being the neurologist that they meet twice a year in the outpatient clinic for their ataxia and baclofen pump refill. The reasons and interests in the research topic were explained to the participants before the start of the study.

### Patient characteristics

Four patients (one male and three females) with a median age of 56.7 years (range 51–72) were included. The demographic data of participants are summarized in Table 1. One patient (Nr. 4) had obvious clinical signs and symptoms of a combination of progressive limb ataxia, dysarthria and a spastic paraparesis. However, we were not able to either verify SCAI-III, SCA VI-VII or hereditary spastic paraparesis through genetic tests. His mother and younger brother suffered from ataxia and gait difficulties, but no genetic tests were available in those cases.

**Table 1:** Patient demographic data.

Patient	Gender	Age	Diagnose	Genetic analysis	Disease duration	Intrathechal baclofen (ITB) therapy duration
Patient 1	Female	51	SCA III	75 CAG repeats in *ATXN3* gene	30 years	1.5 years
Patient 2	Female	52	SCA VII	46 CAG repeats in *ATXN7* Gene	22 years	1 year
Patient 3	Female	52	FA	900 GAA repeats in *FTX* Gene	36 years	4 years
Patient 4	Male	72	SCA	-	12 years	6 months

### Procedure

A total of six interviews (face-to-face) with the participants and the interviewer took place at the outpatient Neurology clinic in Linköping and Uppsala. The interviews were held at the clinic where they attend in two cases in agreement with the participants in a quiet room where they felt comfortable and were not disturbed.

In one case (P1) the interview took place in another clinic (Linköping) by one of the authors who had not met the participant before. In one case (P4), the interview was performed in a nursing home where the patient was admitted because of his deteriorated condition.

Two repeated interviews were carried out with participant 2 and 3. The two authors (SGB and AML) prepared a semi-structured interview guide for the purpose of the interview (Table 2). The interview guide was provided after discussion with experts in qualitative research (GF, AML) in the research group. The reason for choosing specific questions rather than generally asked ones was the speech difficulty (dysarthria) of the participants that did not allow for long discussions (the rationale behind making two interviews for two of the participants at different occasions). The questions were based on previous experiences on qualitative research with other patient groups suffering from chronic disease, and on clinical experiences.

**Table 2:** Interview guide.

1. Please introduce yourself and how you live.
2. Tell us about your life and living with ataxia, how does it interfere with your daily life
3. How was your life before you were treated with ITB, what kind of problems did you experience?
4. What about your life as a whole after treatment with ITB?
5. Tell us about the positive and negative effects of the new treatment.
6. Would you tell us about your experiences of ITB therapy and how it interferes with your sleep and pain sensation? What about spasms and body movements, was there any difference?
7. In what way ITB therapy has affected your daily life?
8. What are your thoughts about the future?

Question number 1, 2, and 8 were open-ended questions that let the patients speak freely about their lives. Every time the patient brought up a specific aspect of the disease and her experiences of it, we tried to encourage her to tell us more about the specific thoughts and feeling of it (Would you tell us more? How were your personal experiences?) Regarding ITB therapy, the questions were more specific because it was easier for patients to answer and we also considered the time aspect (not longer than 180 minutes).

The questions were not pilot -tested before the current study. The interviews were conducted by two of the authors (SGB, AML) and started with an introduction. Both authors were female; SGB had no prior experience in qualitative research, whereas AML had previous training and experience. The interviews lasted between 30–90 minutes, depending on the participants’ speech ability, and were audio-recorded. Field notes were made both during and after the interviews.

### Analysis

The interviews were transcribed verbatim, which means that every word, sound and silent period was written down, and documented as a text. The text was then analyzed according to content analysis guidelines by Graneheim and Lundman [19]. First, in order to get a sense of the whole, the transcribed interviews were read through several times. In the next step, meaning units were identified in the text. This step did not follow common grammatical and linguistic rules, and the division was rather based where a shift of meaning could be discerned. The meaning units were in the next step condensed and coded. Codes with similar content were brought together and labeled as categories. Each of these categories reflected the experiences perceived by the participants in the study. Examples of the analytical procedure where participant quotations are presented to illustrate the categories are summarized in Table 3. All authors were involved in the content analysis process with GF as leading and experienced researcher in qualitative research analysis. No software was used to manage the data. The transcripts were not returned to participants for comment and/ or correction. However, the participants have been provided feedback on the findings at their regular visits to the clinic.

**Table 3:** Examples of the analytical procedure.

Text from the interview/ Meaning unit	Condensed meaning unit	Interpretation of the underlying meaning	Sub-category	Category
None of my children or grandchildren has noticed any signs, they know that the chance is there, like my daughter saying: “Mom you are so damn sick”.	None of her children or grandchildren had noticed signs of the disease	Concerns about the future and how the disease will affect her children	Living with a hereditary disease is associated with concerns about the future	Uncertainty about the future
I have regained the control of my body since I got the baclofen pump. I feel more confident when I walk with my walker at home and when I sit in my wheelchair. My legs stay at the same place, they don’t jump or involuntarily kick without warning.	She can control her body movement and is more confident in walking. Her legs don´t kick involuntarily	A sense of regaining the control of the body movements as a whole due to ITB therapy	Controlling body posture and movement by ITB therapy	ITB therapy, positive effects

## Results

The interviews identified one overall theme and five categories, and thirteen subcategories of living with ataxia and ITB therapy, all of which are presented in Table 4. The overall theme that emerged from the analysis was living in the present/ taking one day at a time. This theme covered the main categories, which are exemplified by quotations relating to patients’ experiences (Table 3 and S1 and S2 Tables). The following categories were elucidated: 1) Uncertainty about the future as a consequence of living with a hereditary disease; the disease 2) Impact on life as a whole 3) Influence on personal life in terms of feeling forced to quit employment, and 4) Influence on daily activities, 5) ITB therapy; advantages and disadvantages.

**Table 4:** Overview of the overall theme, the five main categories, and the thirteen subcategories.

Overall Theme	Main categories	Subcategories
Living in the present/taking one day at a time	1. Uncertainty about the future	About making plans for the future
		About heredity of the disease
		About possible transmission to the children
	2. Impact on life as a whole	On normal functions
		On social life
		On mood
		On being a parent
	3. Feeling forced to terminate employment	To stop working
		Lack of support from the society/employers
	4. Limiting daily activities	
	5. Intrathecal baclofen therapy: advantages/disadvantages	Improved control of the body
		Improved sleep quality
		Increased body weight
		Recommendation of the therapy

### 1. Uncertainty about the future as a consequence of living with a hereditary disease

#### 1.1. Uncertainty about making plans for the future

The participants were all concerned about the physical and psychological consequences of living with an untreatable, progressive disease. Being through the rapid loss of physical functions, three of them expressed that they were trying to live for the moment; they would not make plans for the future.

“I know that I have no future, yes, actually, and I realize that somehow I don´t have any history either”

(P1)

#### 1.2. Uncertainty about heredity of the disease

All participants had performed a genetic DNA test before the diagnosis of ataxia was settled (Table 1). One of the participants was aware of having ataxia before the genetic test was done. The result of the test was a confirmation of what she already suspected. There was a difference between participants in how many family members had been diagnosed with ataxia. Participant 3 was the only one affected by the disease in her family, whereas for participants 1, 2, and 4 there were several family members already suffering from ataxia.

“Everyone, it feels like, no but I have been grown up with ataxia, my father had it, my grandmother had it, my younger sister has it and all but one of my grandmother’s siblings had it”.

(P1)

The disease started early, in three cases already during the teenage period and the onset symptoms were gait ataxia, recurrent falls or a feeling of the ground disappearing under the feet. Slurred speech was also mentioned as an early symptom.

#### 1.3. Uncertainty about possible transmission to the children

The choice of becoming a parent or not and the uncertainty about the disease transmission is a major concern in the life of patients living with a genetic disease. The participants were aware of the risk of discovering the symptoms and signs of the disease in their children, but all of them chose to not encourage the children to do genetic tests, but rather left the decision to the children themselves.

“None of my children or grandchildren have noticed any signs, they know that the chance is there, like my daughter saying: “Mom you are so damn sick”.

(P1)

“My son asks me if his chance of becoming ill is bigger than my caregiver. I think he is aware of the risk to become ill, but then I explain to him that he can have a good life anyway. He could test himself, but I left the decision to him.”

(P3)

### 2. Impact on life as a whole

#### 2.1. Impact on normal functions

The participants were asked about the impact of the ataxia on life. They all agreed that the disease affected several important neurological functions necessary for a normal life such as speech, hearing and swallowing function, vision and the ability to read because of eye movement disturbances in addition to reduced motor and sensory functions in arms and legs.

*[Sighs]* “It affects everything, I have no sensibility left in my legs, then I have a pump operated in my abdomen, I need to buy enormous amounts of clothes, I have speech difficulties, reading difficulties, I have stopped riding my bike and jogging and skating”

(P1)

“I am bound to a wheelchair, I’m not able to walk or to coordinate my movements or to swallow properly, and I need to have assistance all my waking hours”

(P3)

#### 2.2. Impact on social life

The disease also affected the social life in terms of meeting with friends and being surrounded by other people. The most frequent complaint was the declining voice and slurred speech, which was experienced as embarrassing. Depression and avoiding social activity such as singing together with friends were the consequences of dysarthria.

“I am afraid that I am not the kind of grandmother I’d like to be, that my granddaughter can’t hear what I am saying, “Sometimes she looks up at my caregiver and asks: *What does my grandma say*? My speech has become worse and that is the worst part of my disease.”

(P2)

“There was a time when I did not want to see my friends, we sang together in a choir, but because of my declining voice I was not able to continue singing. I became more and more isolated, later that year I was diagnosed with severe depression.”

(P1)

In two cases the affected eye movements prevented the patient from reading books that used to be a great pleasures in their lives. The difficulty was in focusing the vision on the sentences and to follow a text, which is a consequence of nystagmus. Thus, giving up interests and habits was experienced as a negative aspect of the disease.

“I can no longer read books because I can not find the next line. I kind of miss it but I have to get used to it, someone else reads and I listen.”

(P3)

“The letters are jumping and it makes it hard to read books.”

(P1)

#### 2.3. Impact on the mood

Apart from the physical disability that the disease brought to the participants, there was a mental process going on at the same time trying to accept and cope with the disease. All participants had been suffering from depression both when the diagnosis of a genetic disorder was confirmed and later in life.

“Years ago at the beginning of my disease I lost my job, a lot of things happened in my life, I got my diagnosis, I was fired and I was in the process of divorce. My whole life changed, yes, back then I was depressed. I went to see a psychologist and according to the test he did I suffered from severe depression.”

(P3)

“Yes, it is terribly depressing. For three years I woke up seeing my own funeral and death announcement.”

(P1)

#### 2.4. Impact on being a parent

Living with a chronic disabling disease not only affects the role of being a parent or partner, but also reflects the expectations of the society. One of the participants expressed her feelings about the low expectations she had met after she had been bound to a wheelchair. It included both expectations from her child and the society. It is important to realize that physical disability especially speech disturbance may be interpreted as intellectual impairment. Despite progressive neurologic dysfunctions in this group of patients, the cognitive functions remain intact.

“When my son fell ill at school, he called his father instead of me, just because I am sitting in a wheelchair.”

(P2)

“When I was healthy, people had high expectations of me, now they are lower. I don’t know if this is specific for me, but on the other hand you are perceived as stupid quite often.”

(P2)

“In the morning when I get up I don’t think to myself: I am ill or that I will be ill the entire day”. I think different thoughts as other people do, but then some people could show up telling me: “how nice that you can get out of the house.” There exists a huge ignorance amongst people; we live completely different lives. “

(P2)

### 3. Influence on personal life in terms of feeling forced to terminate employment

#### 3.1. To stop working

At the time for the study interview none of the participants worked anymore, but all of them were retired. Participant 3 retired at 30 years of age. They had tried different kinds of employments from being a factory worker to more qualified work such as being the financial manager for three regions of a big state-owned company with great responsibility. The participants either felt that they should quit the job by themselves or had been dismissed from work by the employer. They also mentioned the economic consequences of losing their employment.

“I quit my job for another reason six years ago. I decided after a while and after talking to the employment office that I will do, because I knew that I had ataxia and decided to do a genetic analysis…go home and get retired. I couldn’t argue anymore.”

(P1)

One of the participants expressed her feeling of being dismissed.

“It was a big disappointment and a shock.”

(P3)

Unemployment followed shortly after the diagnosis was announced. In one case, the patient both lost her job and was informed of the diagnosis of cerebellar ataxia within three months. This coincidence was extraordinarily painful to bear.

#### 3.2. Lack of support from society/employer

The lack of support from the society and the employers/heads of the company were expressed as a great disappointment. It created a sense of not being useful anymore. In one case the disappointment over losing the job after many years at the same company and not getting any support from the employer was experienced worse than facing the diagnosis of hereditary ataxia.

“Not only did I lose my job, but I lost an illusion; the illusion of getting help, that the head of the company would take care of me. I still go back to work and work furtively during nights. It is now ten years ago, but it was worse than the disease “

(P3)

“I went to see a fellow from the capital city who was supposed to provide job offers. I went to him bringing my CV to see what kind of job offers he had, instead he dismissed me. I was shocked.”

(P3)

“I retired 25 years ago at the age of 30, I sold my house and my car. My husband lost his job, so we ended up selling everything of value to overcome the negative economic consequences of the disease.”

(P1)

### 4. Limiting daily activities

The daily activities were limited because of the disease. The participants must give up some of their previous physical activities as well as reading and writing. In two cases (P1 and 3) the participants kept on finding other ways to be physically active despite the great disability, they experienced in daily life.

“Doing too much exercise gives me terrible cramps, so I try to find a balance between training and cramps. Unfortunately I am not able to ride a bike outside the house, but I’ve talked to the commune authorities to get a training bicycle at home.”

(P1)

“I can still solve the crosswords with help from my assistants, making food or at least asking them to do so, but it is more difficult to write, it’s ataxia, “the pen doesn’t want to.”

(P2)

### 5. Intrathecal baclofen therapy; advantages and disadvantages

The overall experience with the ITB pump was very good in terms of its impact on several domains as follows:

#### 5.1. Improved control of the body

The therapy improved the participants’ control of their body posture and movements.

The absence of spasticity and cramps and a highly improved upright position, as well as lying position during the night, were among positive effects of ITB therapy experienced by all the participants. Involuntary movements with the legs before ITB therapy was a common complaint in all participants.

“I have regained the control of my body since I got the baclofen pump. I feel more confident when I walk with my walker at home and when I sit in my wheelchair. My legs stay at the same place, they don’t jump or involuntarily kick without warning”

(P3)

“I am left with about 10% of my spasticity, which allows me to maintain the muscle volume. I am so happy to be able to sit in an upright position.”

(P1)

“I can now talk to people without getting disturbed by movements in my legs, I manage to hold a food tray in my lap and to sit down in a car without kicking the car panel.”

(P3)

“I can have my grandchild on my lap without being afraid of kicking her off with my legs, I can be like a common grandma.”

(P2)

#### 5.2. Improved sleep quality

Disturbed sleep often related to pain during the night is one of the major indications for ITB therapy. Three of four participants suffered from pain before ITB therapy and they were all satisfied with the positive effects on sleep and long lasting pain.

“I used to roll out of bed and fell down to the floor, I sleep better now with my legs lying still in the bed, and I have fewer cramps in my back.”

(P3)

“My life before the baclofen pump was completely disturbed by painful spasms that increased during the day, and at night I had difficulties falling asleep. Every time I laughed or coughed my legs started cramping and it was painful.”

(P2)

#### 5.3. Increased body weight

Among the negative effects of ITB therapy two participants mentioned weight gain, another one felt that her body has become heavier despite the unchanged weight and her walking ability has become worse. There were also complaints about the pump being placed in the abdomen in a way that made certain activities, such as shopping, more difficult.

“My walking ability has become a bit worse since I got the baclofen pump, but I’d rather live with that than with my legs getting stuck or involuntarily kick.”

(P3)

“The pump takes up a lot of space in the abdomen, so I am a bit disturbed by that, I also have to put on larger clothes in order to hide it. And then when you walk through the supermarket with a shopping cart that rubs against the pump, it hurts.”

(P1)

One of the participants (P4) expressed a sense of dissatisfaction because of rather unrealistic expectations about the effects of baclofen pump therapy. He was expecting that the treatment would improve his gait unsteadiness and ataxia.

“My expectations in getting better with spasms were met, but when it comes to ataxia, I am disappointed. Somehow, I was hoping for a dramatic improvement.”

(P4)

#### 5.4. Recommendation of the therapy

Three participants completely agreed about that they would recommend the intrathecal baclofen treatment to people in the same situation as them.

“I have already recommended this treatment to people in my situation with the same disease. One of them has a lot of pain and is worried. I think everyone should get the treatment; you do much better with the baclofen pump.”

(P1)

## Discussion

In the present study, the participant’s experiences of living with ataxia emerged in one main theme, i.e. living in the present or taking one day at a time. Following categories were covered by the theme: 1) Uncertainty about the future as a consequence of living with a hereditary disease; the disease 2) Impact on life as a whole 3) Influence on personal life in terms of feeling forced to quit employment 4) Influence on daily activities, and 5) ITB therapy, advantages and disadvantages.

All the participants expressed a strong feeling of uncertainty about the future, confirming this important concept being recognized in a number of chronic illnesses [20–22]. Being through a progressive decline of physical functioning they would not make plans for the future, but live for the moment. The process of achieving the diagnosis of ataxia was described as long as in three cases took many years to confirm. This is due to the slow and insidious onset of the disease, especially in the autosomal recessive mode of inheritance, whereas the autosomal dominant traits are often recognized early in the affected families. In terms of reactions to a diagnosis of chronic disease, the participants described depression and psychological distress, supporting previous studies of patients with MS [23, 24]. Despite the uncertainties about the treatment and prognosis, the genetic confirmation of the diagnosis of hereditary ataxia was important for our participants because of the legitimacy that the diagnosis brings on a patient’s symptoms, results in the access to therapy and supportive care and management. A Diagnosis creates both a framework for medical care by applying a label to the patient and a social order that provides status and permission to be ill [25]. Once the uncertainty about the diagnosis was removed, the question of family planning and risk for transmission to future generation maintained the feeling of uncertainty about the future. A study of internet discussion forums by people with ataxia showed that the issue of inheritance was generally avoided and considered troubling in the sense that it was associated with feeling guilty [7]. Accordingly, our patients had not discussed genetic testing with their children.

The disease impact on life included several important domains of which slurred speech interfered directly with self-identity, relationships and social life. This experience supports the results of a qualitative study assessing the psychosocial impact of dysarthria following stroke where the participants expressed a feeling of stigmatization, social isolation, and changes in self-identity [26]. To our knowledge this study is the first to describe the experiences of dysarthria in patients with ataxia and its impact on their life.

Giving up free time interests such as reading because of eye movement disturbances was another consequence of the disease. The comorbidity of ataxia and depression was recognized in all participants in this study. The chronic physical disease is often associated with episodes of major depression [27]. A study of 300 new patients attending general neurological outpatient clinics showed that 47% met the criteria for anxiety or depression according to DSM IV [28]. However, despite the magnitude of the disabilities that our participants struggled with, they had accepted their own limitations and focused on what they did manage from day to day; As a result they decided to live in the present instead of making plans for the future. A desire for acceptance of life and living within one´s limitations and boundaries, and focusing on life’s positive events brought a sense of joy and happiness clearly expressed in a study that assessed MS-related fatigue [29].

The participants experienced stigmatization in terms of lower society expectation after being diagnosed with the disease and wheel- chair bound compared with the time before. The study of SCA3 or Machado-Joseph disease among persons of Azorean- Portuguese heritage in the U.S. and in the Azores Islands explored the nature of the stigmatization based on the theory of social labeling and in this context we found similarities between our patients’ experiences and those in the study [30].

Losing employment and not being offered other work possibilities, and facing a worsened economic situation at the same time or close to the confirmation of the disease by genetic testing was a common complaint from the participants in this study. This is another important aspect of life placed in the category of the stigmatization of people with chronic disease. The patients’ experiences and the economic burden of the disease are in agreement with studies of other chronic conditions where long sickness leave episodes and early retirement were common findings.[29, 31, 32]

The positive effects of the ITB therapy was expressed in terms of improved body position in the lack of involuntary leg movements, better sleep, reduced spasticity, and pain relief. Weight gain, and the pump located in the abdomen brought a feeling of discomfort, but nonetheless, most of the patients were agreed in recommending the ITB therapy to others with hereditary ataxia. This multicenter study includes all available patients with hereditary ataxia in 2017 that have been treated with ITB in Scandinavia. Even though long-term patient satisfaction, efficacy and safety of ITB therapy in spasticity of both cerebral and spinal origin is well documented [33–36], the therapy is still unknown to both patients with inherited ataxia and physicians meeting them. Given the fact that there is no cure for patients with hereditary ataxia it is important to highlight the positive effects of ITB therapy so it can be offered to the patients as a valuable option.

Hereditary ataxia belongs to a category of neurological disease that worsens the patient´s ability to manage over time and leaves the patient with a high burden of disability. The categories reflect the struggle that the participants’ face both in terms of accepting the diagnosis, finding the strategies to manage different physical and mental consequences of the disease, and how a new treatment interferes with their life. We found a good consistency between the presented data and the findings, in which major categories were clearly defined and emerged in the main theme living in the present/taking one day at a time, contributing to the participants´ sense of a meaningful life.

The qualitative method helps us to better understand and report the patients´ experiences of suffering from ataxia in the context of chronic disease. The content analysis process used in this study included reading the transcribed text as many times as necessary to sort out relevant data and the categories. Using interview- guide and the same questions for all participants and collecting data in a limited frame of time reduces the risk of inconsistency. To achieve the credibility in our study we selected representative quotations that cover and elucidate the categories. The readers were provided a few examples of the analytical procedure based on participants’ narratives, which means identifying meaning units, condensation and interpretation of data as well as identifying categories in this study with examples illustrated in Table 3, S1 and S2 Tables.

Another study including the same patients is designed to cover the results of standardized questionnaires measuring the quality of life, physical activity, fatigue, pain, sleep and estimated spasticity and spasm frequency before and after ITB therapy as well as medical reports.

### Conclusions

Our study presents the results of interviews of four patients with ataxia and ITB therapy. Despite the limited numbers of participants in this study, we consider it to be unique. To our knowledge this is the first study describing patients´ experiences with a combination of inherited ataxia and a quite unknown treatment for this group with ITB. Further research with a larger sample is warranted in order to confirm our results.

## Supporting information

S1 File COREQ (Consolidated Criteria for Reporting Qualitative research) checklist. (PDF)

S1 Table Analytical procedure, identifying the main categories and the theme. (DOCX)

S2 Table Analyical procedure, an example of identifying the categories. (DOCX)
