# Supplementary material for: Cerebellar ataxia and intrathecal baclofen therapy: Focus on patients´ experiences
Source: PLoS One. 2017 Jun 27;12(6):e0180054. doi: 10.1371/journal.pone.0180054 (PMC5487051; doi:10.1371/journal.pone.0180054)
Supplement: S1 Table — (DOCX) [file pone.0180054.s002.docx]

**Supplementary Table 1**

| ***Overall Theme*** | ***Living in the present – taking one day at a time*** | | | | |
| --- | --- | --- | --- | --- | --- |
| ***Main Categories*** | ***Uncertainty about the future*** | ***Impact on life as a whole*** | ***Feeling forced to terminate employment*** | ***Limiting daily activities*** | ***Intrathecal baclofen therapy advantges, disadvantages*** |
| **Codes from the interviews**  **P1** | Wanted to do a genetic test to receive the diagnosis, but knew it beforehand | To not be alone, family, other family members | Does not work, quit employment, restricted life | Reduced sensory input,  Troubles breathing, and reduced vision | The position of the pump increased the need for new clothes (-) |
|  | The heredity of the disease results in concern for the children. The children are aware of the risk. | Ataxia affects every aspect of life.  Alternating identity  Become sick from being healthy | Quit the employment after conversing with employment authorities because of ataxia | Has had to give up a multitude of things, but manages some things with assistance | Lost one´s strength (-) |
|  | The children acknowledge and see the disease; there is worry for and amongst the children that they will end up like their mother | Difficult to lose abilities | Quit working,  Tired of arguing  Had to give up working as a result of the disease | A formerly active life | Better posture  Straighter with the treatment  Needed complementary Botox treatment (+) |
|  | Evidently hereditary  Many relatives have ataxia  Feels like every body  Heredity is present in the family  One sister, grand mother and 4 out of 5 siblings had ataxia | Has been depressed,  Imagined one self dead  Took time to overcome | - | Difficulty managing daily tasks  Need of assistance | To be able to stand up straight (+) |
|  | Difficult to see the results of the genetic test  Knew she had ataxia  The test confirmed what she already knew | The whole life is affected by the disease |  | Difficulty speaking, and reading  Speech and voice changed | The location of the pump is restricting (-) |
|  | The disease ´consequences are significant  The future is uncertain  Life restrictions  Feeling of powerlessness for being affected by the disease | Forced to sell horse and car as a result of the disease |  |  | The pump is inconvenient, it hurts and is not aesthetic (-) |
|  | Planning the future;  To be able to ride a bike is the plan for future | To be forced to get rid of everything was hard |  | Find a balance in physical training | Feeling the catheter (-) |
|  | Has a determination to manage life | The body has changed,  Loss of strength  Change- “it is not me” |  |  | Weight gain (-)  Improved sleep (+)  Experiences cramps during the night but manages to go back to sleep (+) |
|  |  |  |  |  | Cramps, clenches teeth, cramps almost at the same time during the night |
|  |  |  |  |  | Before the pump-impaired sleep |
|  |  |  |  |  | Relaxed muscles (+)  Less pain (+) |
|  |  |  |  |  | Mentally challenging  Must fight the body |
|  |  |  |  |  | The remaining functions unchanged: urinating, abdomen, sexual function |
|  |  |  |  |  | The pump is in the way when riding, it hurts (-) |
|  |  |  |  |  | Decreased spasticity, but still does not feel comfortable |
| ***Categories*** | ***Uncertainty about the future*** | ***Impact on life as a whole*** | ***Feeling forced to quit employment*** | ***Limiting daily activities*** | ***Intrathecal baclofen therapy pros and cons*** |
| **Codes from the interviews**  **P2** | The only family member with ataxia, all other members are healthy | Feels sad about not be able to fulfil the role as grand mother  Feeling of not be good enough | Stop working | Difficulty using the arms  Is able to eat | Had a lot of pain and cramps  Treatment with the pump has removed the pain (+) |
|  | Has been sick for 35 years | Slurred speech because of the disease  Difficulty speaking with the grand children |  | The speech is affected, its tough, does not want to speak, avoids speaking | Has stopped taking analgesic medications as a result of pump treatment (+)  Theme: ”independent of analgesic”, a feeling of freedom |
|  | Not sick, has a disability | The grand child gives joy  Plays with the grand child |  | Lost ability to walk | Negative effects of the pump  Theme: weight gain (-) |
|  | Received the wrong diagnosis first, which mad her decide to have children, has one child and one grand child | The wheel chair is limiting  Can not go home to her daughter |  | Lost her ability to write, but cooks food and bakes | Experiences improved sleep, no pain, no need for analgesic medications (+) which made her speech even more slurred  The cramps affected the sleep, but no she is no longer in pain |
|  |  |  |  | Early onset of disability  Walked unsteadily | The pump reduces the cramp frequency and spasticity (+) |
|  |  |  |  | Speech difficulties | The pump does not affect the natural functions such as urinating or defecation |
|  |  |  |  | Mourning the loss of functions | The pump has changed her life |
|  |  |  |  |  | Due to the pump she is able to play with her grand child  To have the child in her lap |
|  |  |  |  |  | Recommends the pump to others |
| ***Categories*** | ***Uncertainty about the future due to ataxia*** | ***Impact on life as a whole*** | ***Feeling forced to quit employment*** | ***Limiting daily activities*** | ***Intrathecal baclofen therapy pros and cons*** |
| ***Codes from the interviews***  ***P3*** | Has had ataxia for 12 years, the diagnosis was made through genetic test  Ataxia is present in several family members  The mother and uncle have the disease | Dependent on assistance | Has worked as an economist  Retired for 9 years | Lost physical abilities such as walking, coordination of movements  Swallowing difficulties | The treatment affects the legs in a way that they stay still in the bed  Does not fall out from the bed |
|  | The symptoms began several years before the diagnosis was made | Walking will be a problem in the future  Obstacles in society | Lost the job and did not get any help from the employer  Feels disappointed about the society´s help in difficult situations | Speech difficulties | The body was more difficult to control before the pump  Uncontrolled kicking of legs |
|  | Living alone  Has a healthy son | Considers herself as a human being  Has a disease  Is a human rather than the disease itself | Was head of the economy department (Samhall) | Deteriorated successively  First cane, then crutches and now wheel chair | Experience of heavier body (+)  Difficulty moving |
|  | Focuses on what she is able to do | Distances herself from the disease | Disappointed by her employer | Walks with a walker inside the house | The body is relaxed, but heavier |
|  | Solves problems, does not look back | Get stressed easily, which affects the mood  Mood swings, gets outbreaks | Felt useless  Disappointed | Hobbies  Shooting with air guns  Can not read by herself | Good sleep (+)  No pain (+) |
|  | Willingness to exercise | Whole life changed at the moment she received the diagnosis  Was depressed | Felt tricked and was shocked by the answer | Fell down when she tried to ride a bike | The spasticity affected a great deal |
|  | Has goals  Rarely looks back | Received help for the depression | A lot of things happened all at once  Unemployed  The disease | Husband left | Better quality of life with the treatment (+) |
|  | Living in present | Two sides of the disease  Expectations become lesser, acceptance becomes higher because of the illness | Maternity leave was the reason why she lost the job, but had also got the diagnosis | Gardening  Sits where she sits | The pump improves, simplifies |
|  | Focuses on the present  Is satisfied |  |  | Goes to the theatre and cinema  Member of a literature circle | It was important to test the pump |
|  | Lives in the present | Lives her life |  |  | Uncontrolled body movement |
|  | More difficult taken into account the son  Does not give up  Is present anyway | Important to feel solidarity  Feel normal and accepted  Meet peoples’ prejudices |  |  | The Body is heavier with the pump (-) |
|  | Difficult losing the partner  Sorrow | Ignorance behind other peoples’ attitudes |  |  | Heavier to move (-) |
|  | Positive answer that ataxia is an unusual disease  Was advised to live, which felt good | Is not perceived as a person but as her disease |  |  | The legs are still |
|  | Divorced as a result of her disease | No positive experiences of the disease |  |  | Reduces involuntary movements (+) |
|  | Tries to remind herself that there are others who have it worse  Get perspective | Can not be a “real mother” |  |  | More tired with the treatment, sleeps more |
|  | Has no plans for the future at all | The son refuses to call her mother,  Wants to protect her in certain situations |  |  | Less spasticity (+)  The ability to have something in your lap |
|  | Takes one year at the time | Sometimes like a ordinary mother |  |  |  |
|  | The son is a cause of concern |  |  |  |  |
|  | Worried about inheriting ataxia |  |  |  |  |
|  | Worried about the son getting ataxia as well  He understands that there is a risk |  |  |  |  |
