# Supplementary material for: Cerebellar ataxia and intrathecal baclofen therapy: Focus on patients´ experiences
Source: PLoS One. 2017 Jun 27;12(6):e0180054. doi: 10.1371/journal.pone.0180054 (PMC5487051; doi:10.1371/journal.pone.0180054)
Supplement: S2 Table — (DOCX) [file pone.0180054.s003.docx]

**Supplementary Table 2**

| **Meaning units** | **Condensed meaning units** | **Codes** | **Categories** |
| --- | --- | --- | --- |
| Are your children healthy?  None of them have noticed anything, but they are aware of the risk, it was my daughter who said:  Mom you are so (.) sick. | P1’s children are healthy, they have not noticed anything but know of the risk and know that P1 is very sick | 1. The heredity of the disease worries the children, they are aware of the risk  2. The children acknowledge the disease, there is a worry for and amongst the children of becoming like their mother | Uncertainty about the future |
| And the family?  Everyone, it feels like, no, but I have grown up with the ataxia, my father had it born in 1937 and my grandmother had it | P1 has many relatives who have/have had ataxia, has grown up with the disease, many in the family have it, enough to where it feels like everyone. An evident heredity on the paternal side, several of the grandmothers siblings had it | 1. Evident heredity, many in the family have ataxia, feels like everybody, heredity is in the family, one sister, grandmother and 4/5 siblings of the grandmother had ataxia  2. Knowledge of the disease and its consequences, uncertain future feels powerless as a result of the disease |  |
| And so I got this diagnosis, it was too much, I knew I had ataxia since the age of twenty, but to have it confirmed, then it was so | Even if P1 knew that she had ataxia already at 20 years old, the confirmation of the diagnosis meant a great deal of stress | Difficult to receive the result of the genetics test, even though she knew she had ataxia, the test just confirmed what she already knew |  |
